# Population pharmacokinetic model of ivermectin in mass drug administration against lymphatic filariasis

**DOI:** 10.1371/journal.pntd.0011319

**Published:** 2023-06-01

**Authors:** Abdullah Alshehri, Yashpal S. Chhonker, Veenu Bala, Constant Edi, Catherine M. Bjerum, Benjamin G. Koudou, Lucy N. John, Oriol Mitjà, Michael Marks, Christopher L. King, Daryl J. Murry

**Affiliations:** 1 Clinical Pharmacology Laboratory, Department of Pharmacy Practice and Science, College of Pharmacy, University of Nebraska Medical Center, Omaha, Nebraska, United States of America; 2 Department of Clinical Pharmacy, College of Pharmacy, King Khalid University, Abha, Saudi Arabia; 3 Centre Suisse de Recherche Scientifique en Côte d’Ivoire, Abidjan, Côte d’Ivoire; 4 Center for Global Health and Diseases, Case Western Reserve University School of Medicine, Cleveland, Ohio, United States of America; 5 Université Nangui Abrogoua, Abidjan, Côte d’Ivoire; 6 Liverpool School of Tropical Medicine, Pembroke Place, Liverpool, United Kingdom; 7 Barcelona Institute for Global Health—University of Barcelona, Barcelona, Spain; 8 School of Medicine and Health Sciences, University of Papua New Guinea, Port Moresby, Papua New Guinea; 9 National Department of Health, Port Moresby, Papua New Guinea; 10 London School of Hygiene and Tropical Medicine, London, United Kingdom; 11 Hospital for Tropical Diseases, London, United Kingdom; 12 Division of Infection and Immunity, University College London, London, United Kingdom; 13 Veterans Affairs Research Service, Cleveland Veterans Affairs Medical Center, Cleveland, Ohio, United States of America; 14 Fred and Pamela Buffett Cancer Center, University of Nebraska Medical Center, Omaha, Nebraska, United States of America; University Hospital Bonn: Universitatsklinikum Bonn, GERMANY

## Abstract

**Background:**

Ivermectin (IVM) is a broad–spectrum anthelmintic drug used to treat diseases caused by filarial worms, such as onchocerciasis and lymphatic filariasis (LF). IVM is part of a triple–drug therapy used by the Mass Drug Administration (MDA) as a preventive strategy to eradicate LF in sub–Saharan Africa. The drug shows high variability in drug exposure in previous pharmacokinetic studies. This study aims to build a population pharmacokinetic (PopPK) model to identify and quantify the possible sources of the variability of IVM exposure after a single–oral dose in LF–infected subjects and healthy individuals.

**Methodology / Principal findings:**

In this analysis, 724 samples were collected from treatment–naïve *Wuchereria bancrofti*–infected (*n* = 32) and uninfected (*n* = 24) adults living in Côte d’Ivoire who had received one dose of IVM as a part of triple–drug therapy. PopPK analysis was conducted using Phoenix NLME 8.3 software. The Monte Carlo simulation based on the final model was performed to simulate drug exposure among different dosing groups (200 μg/kg, 18 mg, and 36 mg). A two–compartment model with zero–order dose input into the absorption compartment with a lag time function followed by first–order absorption and linear elimination best described the IVM’s pharmacokinetic (PK) parameters. The final model identifies that the PK parameters of IVM are not affected by LF infection. Sex was a significant covariate on the peripheral volume of distribution (Vp/*F*, 53% lower in men than in women). IVM drug exposure shows linear pharmacokinetic behavior among the simulated dosing groups with similar drug exposure based on sex.

**Conclusion/Significance:**

We have developed a PopPk model to describe and identify possible sources of the variability of IVM exposure. To our knowledge, this is the first PopPK study of IVM in patients with LF.

**Trial registration:**

NCT02845713; NCT03664063

## Author summary

Ivermectin has been shown to be a valuable tool in mass drug administration campaigns against lymphatic filariasis. The drug shows high variability in drug exposure in previous pharmacokinetic studies. Variability in drug exposure can lead to either a higher exposure with the potential for toxic effects or a lower exposure that may result in treatment failure. Identifying and quantifying the source of high variability using non–linear mixed effects modeling is crucial for future dosing recommendations that seek to maximize therapeutic benefit while minimizing toxicity risks. In this study, the variability of IVM exposure after a single oral dose was investigated with a population pharmacokinetic model using data from Lymphatic filariasis infected subjects and healthy individuals.

## Introduction

LF is a mosquito-borne disease caused by nematode parasites: *Wuchereria bancrofti*, *Brugia malayi*, and *Brugia timori*. After infection, nematodes reside and proliferate in the lymphatic system, where they evoke host immune response and compromise the lymphatic system’s function, which leads to clinical manifestations of the disease, such as lymphedema [1]. The disease’s progression is associated with increased morbidity, such as disability, and social stigma among patients, thus making them unable to function well in their communities [2]. In 2000, the WHO established the Global Program to Eliminate Lymphatic Filariasis (GPELF) to eradicate the disease from endemic areas by 2020 [3]. Based on the current progress in eradicating efforts, WHO has proposed a new target to eliminate the infection from all remaining endemic areas by 2030 [4]. The program is based on Mass Drug Administration (MDA) once or twice a year for all eligible persons in endemic areas to decrease the prevalence of infection in the community to a level that cannot sustain transmission. One treatment protocol that shows high potential for suppressing microfilaria load in patients with LF is a combination of three anti-filarial drugs (ivermectin, albendazole, and diethylcarbamazine) known as IDA. The IDA treatment protocol has shown efficacy against LF infection by the sustained suppression of microfilaremia in residents of West Africa and the South Pacific [5–7].

IVM, a broad-spectrum anthelmintic drug, was approved by the FDA for human use almost four decades ago. However, PK studies are scarce in humans, particularly those related to PopPK. PopPK studies have some advantages over traditional PK studies; for instance, the nonlinear mixed-effects model methodology used in PopPK studies enables identifying and quantifying the effect of multiple factors (covariates) to further explain the variability in drug exposure to optimize the drug therapy [8–11]. We have previously shown IVM has substantial PK variability, with systemic drug exposures (AUC) ranging over 10-fold when administered as a fixed dose based on weight [6]. PopPK modeling of IVM is needed to evaluate dose rationale and the impact of factors associated with observed PK variability. Previous PopPK studies of IVM have examined the effect of different covariates, such as age, weight, and height, on PK parameters of the drug by using data from healthy subjects [12–14]. However, the effect of LF on IVM drug exposure has not yet been examined with the population analysis method using data from patients with LF. The physiological and pathological changes associated with LF might affect drug exposure due to the compromised function of the lymphatic system. The lymphatic system can play an essential role in drug PK; for example, oral drug absorption into systemic circulation can occur through the lymphatic system in the intestine [15]. The compromised lymphatic system further warrants extensive PK studies in diseased subgroups. In MDA against LF, IVM is dosed based on body weight at 200 μg/kg [16]. IVM is a broad therapeutic index drug with a dose of up to 2000 μg/kg that seems safe without significant side effects [17]. Dosing IVM as a single fixed dose could be more practical from logistic concerns in MDA than dosing based on body weight. Fixed single dose at 18 mg or 36 mg using experimental 18 mg tablet has shown linear PK behavior by increasing dosing with similar side effects to weight-based dosing [18].

This study aims to build a population pharmacokinetic model to identify the possible impact of filariasis infection on IVM PK after oral dosing, along with other covariates, to explain IVM exposure variability and use the model to simulate IVM drug exposure in MDA eligible populations after different dosing. To our knowledge, this is the first PopPK study of IVM in subjects infected with *Wuchereria bancrofti*.

## Methods

### Ethics statement

Written informed consent was received from the study participants before beginning the study. Institutional review boards in Cleveland, USA (University Hospitals Cleveland Medical Center IRB #03-16-09) and Côte d’Ivoire approved the study protocol (Comité National d’Ethique et de la Recherche, CNER, N/Ref: 022/MSLS/CNER-kp). This trial is registered at clinicaltrials.gov (NCT02845713).

### Clinical data

The data from a recently published IDA therapy study was used to build the PopPK model [6]. The study was an open-label cohort study assessing the PK, safety, and efficacy of single-dose triple-drug therapy (ABZ, DEC, and IVM) in adults with and without *Wuchereia bancrofti* infections in the Agboville district of Côte d’Ivoire. PK data from naïve treatment infected and healthy adults were used in the PopPK model-building process. The eligibility criteria for study participants were as follows: adults aged 18–70 years without a prior history of acute illness and who had not received ALB or IVM in the past two years. Participants with a LF history were eligible for the study if they had a microfilaria plasma level of 50 Mf/ml or above. Participants were excluded if they had a history of renal or liver disease, a serum alanine transaminase, aspartate transaminase, or a creatinine level more than 1.5 times the upper limit of normal or blood hemoglobin less than 7gm/dl. Women were also excluded if they had a positive pregnancy test. Individuals who had taken medication within one week of study onset that could interfere with the metabolism of the study drugs were also excluded. Individuals were excluded if they had a urinary tract infection (UTI) confirmed by finding more than 10 neutrophils in a urine sample or 3+ nitrate on a dipstick.

### Study design and bioanalysis

Participants were administered a single dose of IVM (200 μg/kg), DEC (6 mg/kg), and ABZ (400mg) after a high-fat breakfast. Plasma samples were collected before dosing and at 1, 2, 3, 4, 6, 8, 12, 24, 36, 48, and 72 hours and 7 days after treatment. Plasma samples were isolated by centrifugation, and plasma aliquots were stored at -20°C until analysis. The plasma concentrations of three drugs were analyzed by the previously described validated liquid chromatography-mass spectrometric (LC-MS/MS) methods [19–21].

### Population pharmacokinetic analysis

PopPK analysis was conducted using nonlinear mixed-effects modeling with Phoenix NLME (version 8.3; Certara L.P., St. Louis, MO, USA) software. The first-order conditional estimation method with interaction (FOCE-I) was used in the model-building process. The model-selection process was guided by the following: 1) the minimization of the objective function value (OFV), 2) the visual inspection of the goodness-of-fit plots, 3) the reduction of interindividual variability (ω^2^) and residual variability (σ^2^), and 4) the estimation precision of the population parameters (CV%). The goodness-of-fit plots include observed plasma concentrations versus individual predictions (IPRED) and population predictions (PRED), conditional weighted residuals (CWRES) versus time after the dose, and population predicted concentrations (PRED).

### Base model

**Fig 1:**
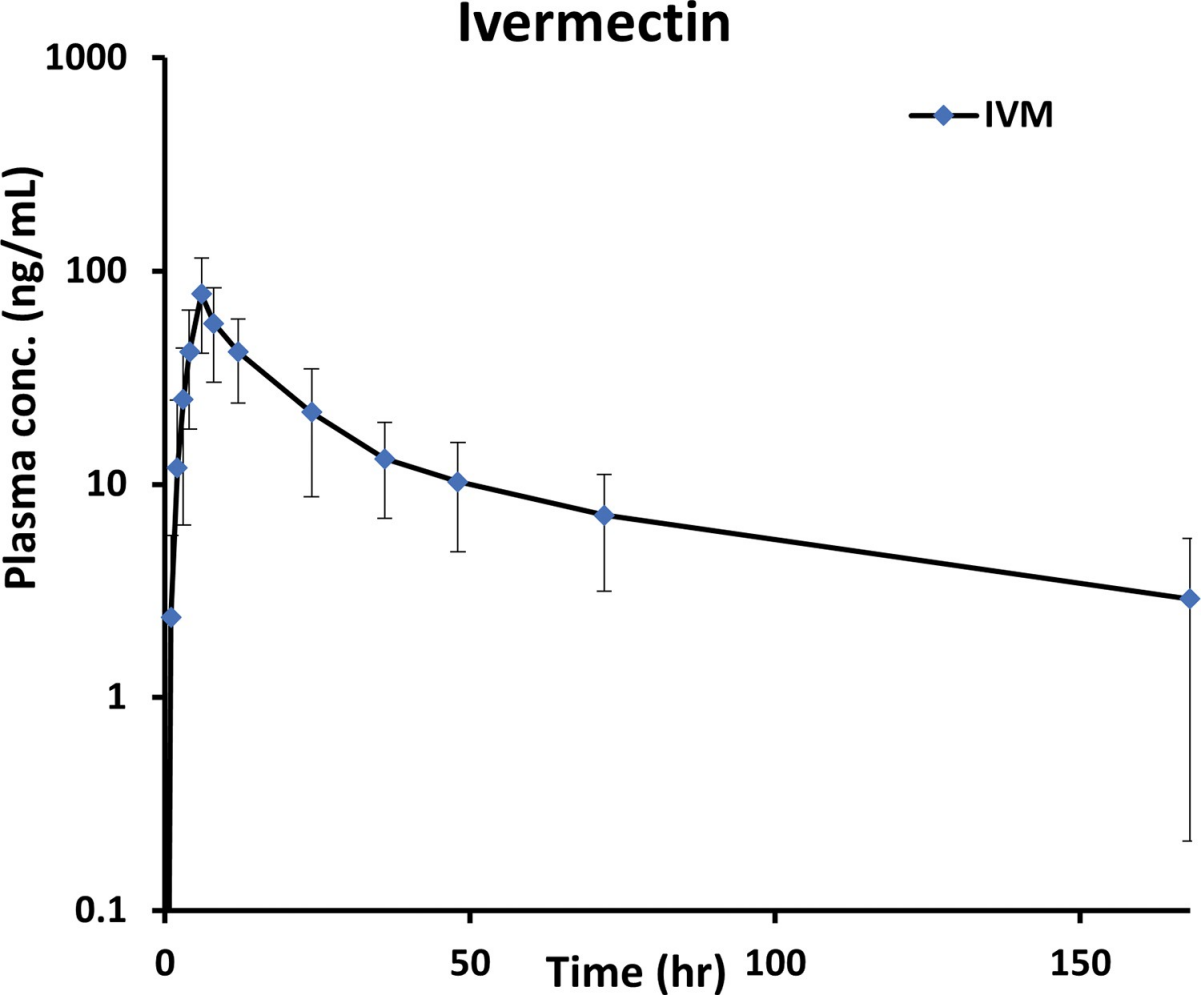
IVM plasma concentration vs. time after 200 μg/kg dosing (n = 56).

The plasma concentration-time curve was used first to visualize the data (**Fig 1)**. Different structural models with one or two compartments were evaluated to find the best model to fit the data. Several absorptions models were explored to describe IVM absorption, including absorption lag time, transit compartments, and different mixed first and zero-order absorption scenarios. The initial estimation of PK parameters used in the model building process was performed from the original dataset using the Phoenix WinNonlin software (version 8.3, Pharsight Corporation, USA) non-compartmental analysis (NCA) tool. Interindividual variability (IIV) in estimated PK parameters was assumed to be log-normally distributed and explored using the exponential relationship between PK parameters and tested covariates. Residual variability was evaluated using different error models, including additive, proportional, and combined additive-proportional error models.

### Covariate analysis

First, the possible effects of covariates on the variance of inter-individual variability between subjects in PK parameters (ETA) were visualized using ETA plots. Covariates evaluated were sex and infection status as categorical variables and age, weight, creatine level, ALT, and AST as continuous variables. Creatinine clearance (CrCl) was not included in the covariate analysis since IVM is mainly eliminated into feces with negligible renal excretion [22]. Allometric scaling using the average body weight was used to standardize the weight effect on PK parameters. Fixed exponent values of 1 and 0.75 were applied to the apparent volume of distribution and clearance of central and peripheral compartments, respectively. The relationship between continuous covariates and PK parameters was added to the model using the following equation:

$$Pi=P_{tv}\times {\left(\frac{\mathrm{cov}\;i}{COVp}\right)}^{\mathrm{Cov}.E}\times \exp\;(\eta \;Pi)$$ (1)

Where *Pi* is the parameter value of the *i*th subject, *P*_*tv*_ is the typical parameter mean value in the study population, *COVi* is the covariate value of the *i*th subject, *COVp* is the covariate population average, *Cov*.*E* is the covariate effect, *ηPi* is *i*th subject’s deviation from the typical population value.

For categorical covariate, the relationship was added using the following equation:

$${\mathrm{Pi}=P}_{\mathrm{tv}}\times \exp(\mathrm{Cov}.E*\mathrm{Cov}.\mathrm{cat})\times \exp(\eta \;\mathrm{Pi})$$ (2)

Where *Cov*.*cat* is categorical covariate value of 0 (female sex or -ve infection status) or 1 (male sex or + ve infection status).

Covariate selection for the final model was performed using a stepwise forward addition followed by backward elimination. In forward addition, a covariate is considered significant if its addition to the selected base model decreases OFV by more than 6.635 (P < 0.01) or increases OFV by more than 10.82 (P < 0.001) in the backward elimination step [23,24].

### Model evaluation

GOF plots and estimation precision of the population PK parameters from the final model were used to compare the fitting improvement of the final model compared to the base model after incorporating the effect of significant covariates into the selected final model.

### Model validation

The validity of the selected final model was evaluated using the following approaches:

- **VPC**_**S**_: The prediction performance of the final model was assessed by a visual predictive check (VPC) with 1,000 replicates. The model-based 95% prediction interval of simulated concentrations at the 5th, 50th, and 95th percentiles were compared to the same percentiles of observed concentrations.
- **Non-parametric bootstrap:** the stability of the final model was assessd by bootstrap method. 1000 datasets were created by resampling with replacements from the original dataset, and PK parameters medians and confidence interval estimated by bootstrap were compared to those estimated by the final model.
- **External validation:** Two hundred eighty-six new IVM plasma concentrations from 25 participants were used to validate the final model externally. The data were from a recently published randomized, open-label clinical trial to investigate PK interaction between azithromycin and IDA therapies [25]. The study subjects received the same dose of IVM (200 μg/kg) as the index data. The plasma samples were collected before dosing and at 1, 2, 3, 4, 6, 8, 12, 24, 48, and 72 hours. Plasma samples were analyzed using the previously described validated liquid chromatography-mass spectrometric (LC-MS/MS) methods [19–21]. A visual predictive check with 1,000 replicates was performed after fixing structural and error parameters estimated by the final model.

### Simulation

A simulation based on the final model was conducted to simulate and compare the drug exposure estimates following different single-dose regimens. The Monte Carlo simulation was performed using the final model parameters with IIV to predict PK profiles for 1000 virtual subjects in each dosing group (200 μg/kg, 18 mg, and 36 mg). Simulated body weights were sampled from a normal distribution with a sex ratio similar to the demographic characteristics observed in the parent clinical study. The area under the concentration-time curve from time zero to the last measurable concentration (AUC_0-t_) and peak serum concentration (C_max_) were estimated to compare the drug exposure across simulated dosing groups.

## Results

**Fig 2:**
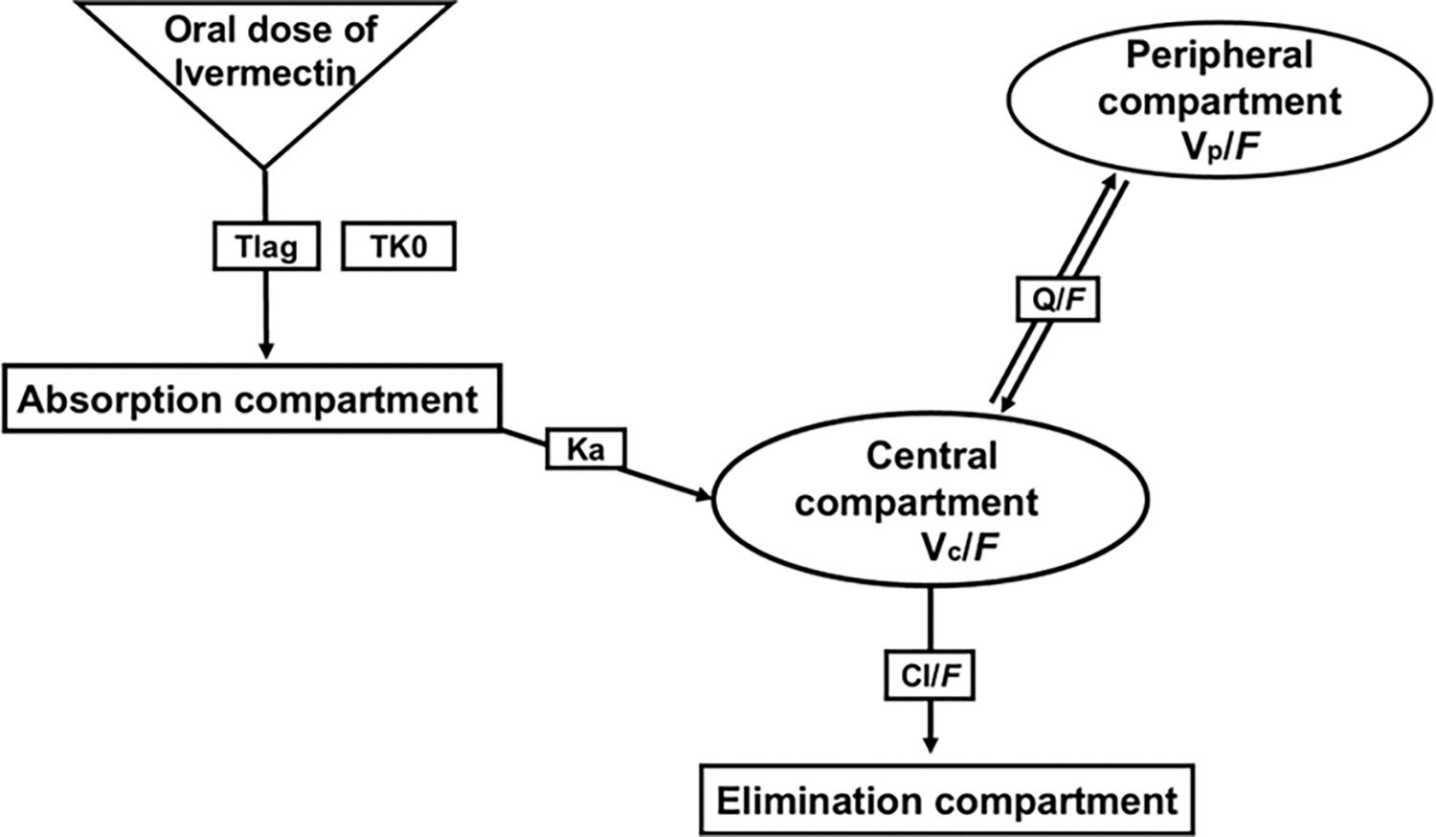
Schematic description of the selected population structural model. Tlag, lag time function; TK0, zero dose input into absorption compartment; Ka, first–order absorption rate constant; VC/F, central volume of distribution; Vp/F, peripheral volume of distribution; CL/F, clearance; Q/F, intercompartmental clearance.

**Table 1** summarizes the study subjects’ demographics and clinical characteristics. Of 56 participants, 32 were infected with *Wuchereria bancrofti*, and 24 were uninfected. A total of 724 plasma samples were used in the model-building process. Plasma concentration of IVM at 168h was missing for four participants due to not showing up for the last plasma sampling. All measured plasma concentrations were above LLOQ (0.1 ng/mL). The performance of the different structural models was assessed based on the goodness-of-fit criteria. **S1 Table** shows OFVs for the tested structural models. A two-compartment model with zero-order dose input into the absorption compartment with a lag time function followed by first-order absorption and linear elimination best described the IVM disposition. **Fig 2** shows a schematic description of the selected structural model. The combined additive-proportional error model best explained the residual variability. The visual inspection of the goodness-of-fit plots for the base model showed a satisfactory fit for the structural base model **(S1 Fig)**. The initial evaluation of the covariates’ effects on the ETA value of the PK parameters showed that sex was impacting the ETA value of the apparent volume of distribution (Vp/*F*) of the peripheral compartment, with females showing a higher median ETA value than males **(S2 Fig)**. Other tested covariates did not show any trend in the ETA value distribution of PK parameters.

**Table 1:** Subjects demographics and clinical characteristics.

Demographic and clinical characteristics	Value
**Sex**	
No. of males (%)	32 (57%)
No. of females (%)	24 (43%)
**Infection status**	
Lymphatic filariasis—ve (%)	24 (43%)
Lymphatic filariasis + ve (%)	32 (57%)
**Age** (yr) Median (range)	40 (18–66)
**Weight** (kg) Median (range)	61.6 (51–135)
**ALT** (u/L) Median (range)	25 (14–67)
**AST** (u/L) Median (range)	29 (15–53)
**Serum creatinine** (mg/dL) Median (range)	1.1 (0.6–1.6)
**WBC** (10^6^/L) Median (range)	6.5 (3.3–9.8)
**Hg** (g/dl) Median (range)	12.85 (8.8–16.4)

(ALT), Alanine Transaminase; (AST) Aspartate Aminotransferase, (WBC) White Blood Cells, (Hg) Hemoglobin

However, all covariates were included in the forward and backward stepwise selection process, and sex was the only covariate from the tested covariates to decrease the OFV, showing a statistically significant impact on the Vp/*F*. After incorporating the effect of sex on the Vp/*F* in the final model, the non-diagonal covariance model “full block” was tested to determine if it could improve the fitting further by minimizing OFV. A model with non-diagonal covariance improved the model fitting by minimizing OFV (ΔOFV = -144) compared to the diagonal model (ΔOFV = -28). Based on this, a model with non-diagonal covariance incorporating the impact of sex on Vp/*F* was selected as the final model. After including the effect of sex in the final model, the following equation describes the impact of sex on IVM Vp/*F*:

$$Vp/F_{i}=424.3.e^{‐0.74\times \mathrm{sex}}$$ (3)

where Vp/*F*_*i*_ is the individual value of the apparent volume of distribution of peripheral compartment.

**Fig 3:**
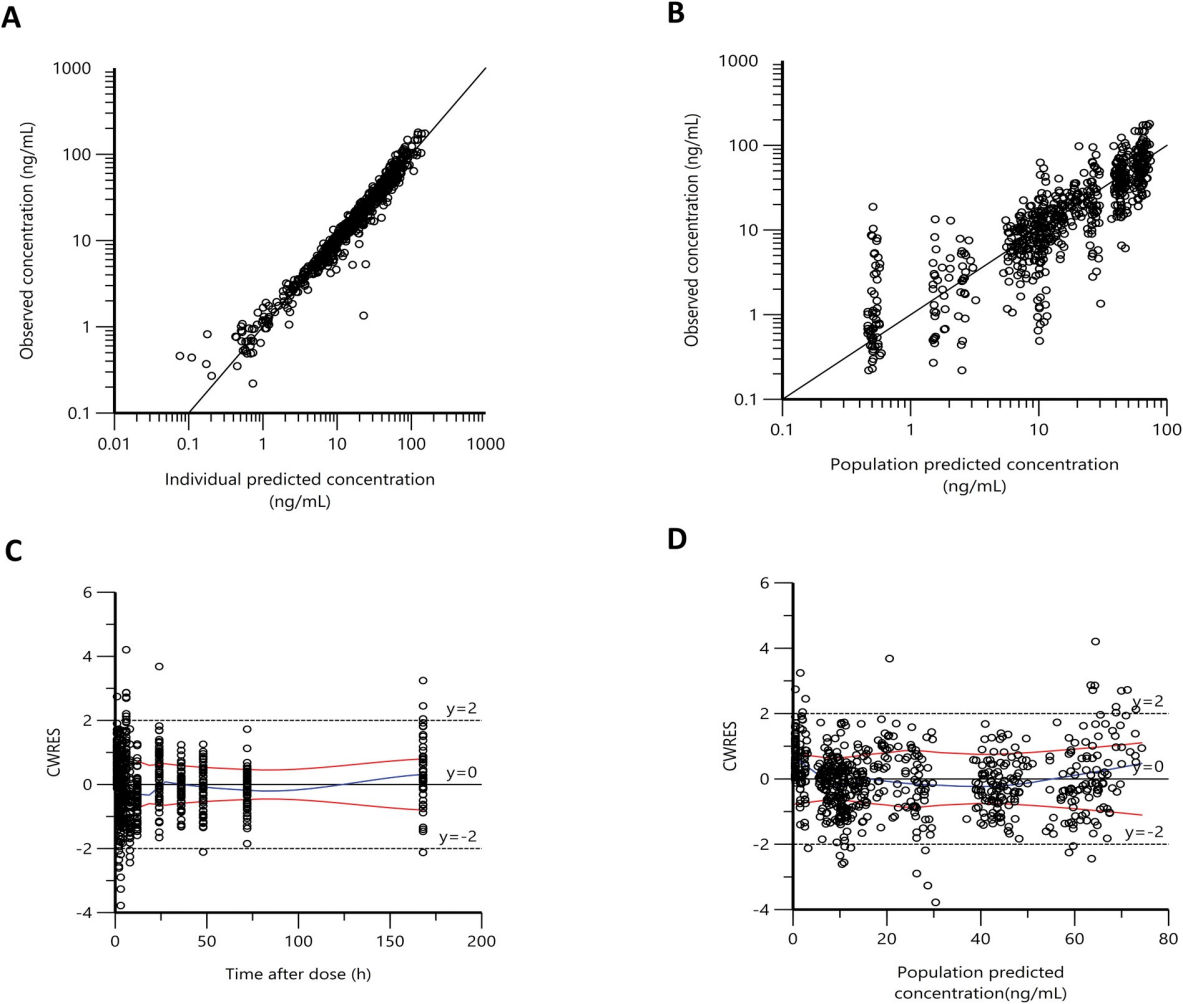
**The goodness of fit plots of final model** Observed plasma concentrations of IVM versus individual predicted concentration (A) and population predicted concentration (B), conditional weighted residuals (CWRES) versus time after dose (C) and population predicted concentration (D).). Black lines in (A) and (B) are the line of identity. The blue line in (C) and (D) represent the locally weighted scatterplot smoothing line (LOWESS); dashed black lines are margins (y = 2) of outliers.

**Fig 4:**
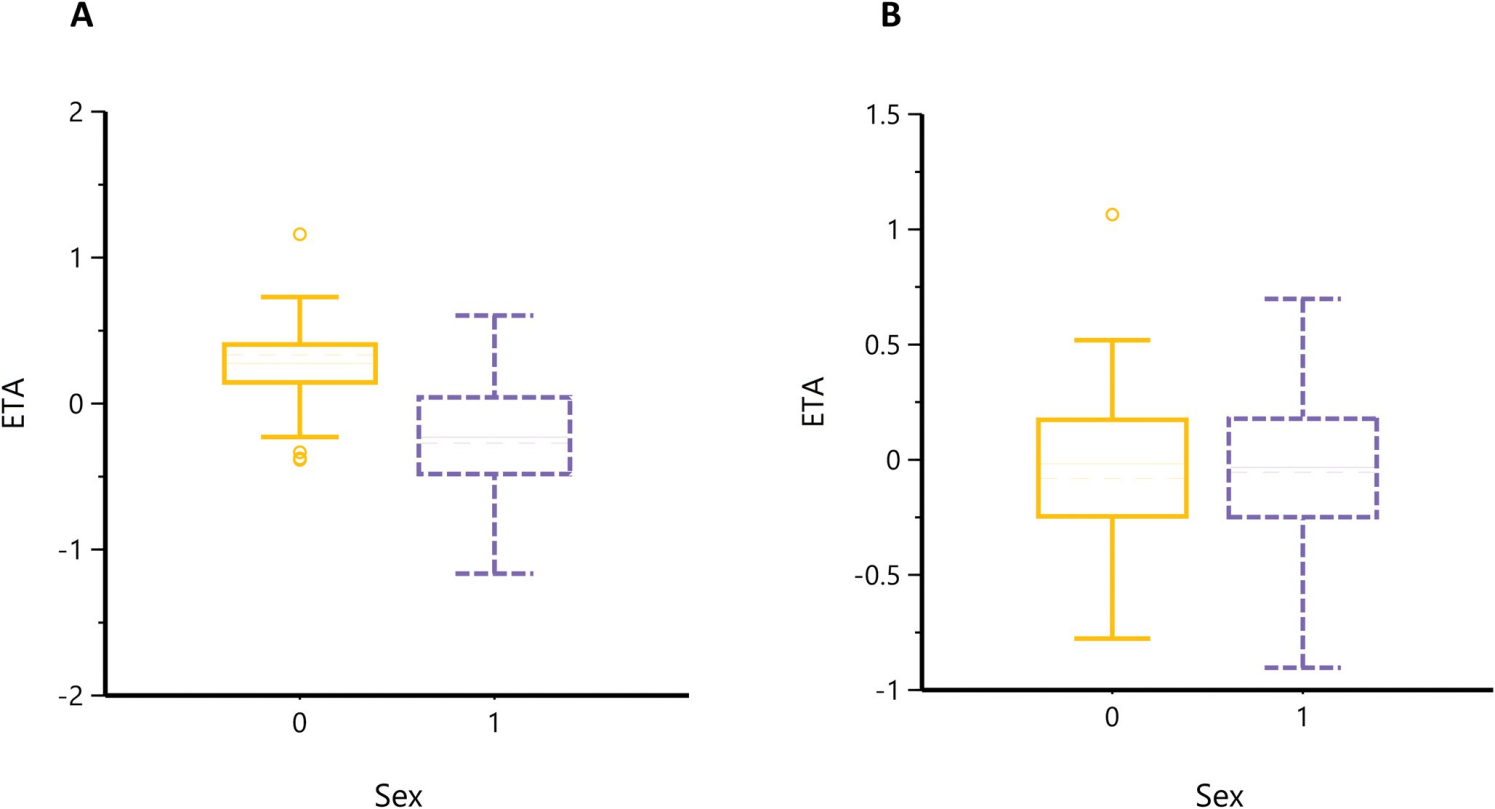
ETA box plots for sex impact on Vp/*F*. Box plots show the impact of sex on Eta values of Vp/*F* in the base model (A) and after incorporating the sex effect in the final model (B). (0 = Female, 1 = male).

The final goodness-of-fit plots showed improvement in the fitting of the final model compared to the base model (**Fig 3)**. The observed plasma concentrations vs. IPRED and PRED plots show a good distribution of values on either side of the identity line (**Fig 3A and 3B**). Residual diagnostic plots of error distribution showed that most CWRES were distributed between 2 and -2. The distribution was symmetrical on either side of the zero line over the predicted concentration range, and the time after dosing indicated a satisfactory fit with no apparent pattern in the error distribution of the final model **(Fig 3C and 3D)**. The final model estimation of the PopPK parameters (the fixed-effect parameters) and the interindividual variability (the random-effect parameters) with the coefficient of variation (CV%) are shown in **Table 2**. Estimates of mean population values for Vc/*F*, CL/*F*, Q/*F*, Ka, TK_0_, and Tlag were 138 liters, 7.02 liters/h, 9.11 liters/h, 0.71 1/h, 3.7 h, and 0.75 h, respectively. The model estimation of Vp/*F* was 424.3 liters in women and 200.4 liters in men. The final model shows a good precision in estimating fixed-effect parameters with CV% less than 14%. The effect of sex on Vp/*F* included in the final model decreased IIV in Vp/*F* by 43% from 0.30 to 0.17 compared to the base model. Moreover, it corrected the observed trend in the ETA values distribution of (Vp/*F*) seen in the base model (**Fig 4).** Shrinkages in IIV for estimated compartment parameters (CL/*F*, Vc/*F*, Q/*F*, Vp/*F*) were less than 15% **(Table 2).** The low percentage of shrinkage indicates that the data used to build the model are informative in explaining IIV. The shrinkage in the epsilon value was 18%.

**Table 2:** Final model estimations of population PK parameters of IVM.

Parameter	Estimate (CV %)	Shrinkage (%)	Bootstrap Median Value (2.5th-97.5th percentiles)
**Fixed–effect parameters**			
CL/*F* (L/h)	7.02 (6.7)		7.01 (6.17–7.95)
Q/*F* (L/h)	9.11 (10.4)		9.04 (7.43–10.88)
V_c_/*F* (L)	138 (8.4)		137.17 (117–159.50)
V_p_ /*F*(L)	424.33 (8.4)		437.94 (368.45–530.87)
Tlag (h)	0.75 (9.5)		0.76 (0.63–0.93)
Tk_0_ (h)	3.73 (5.3)		3.73 (3.33–4.11)
Ka (1/h)	0.71 (13.6)		0.72 (0.53–0.99)
Sex (male) on V_p_/*F*	-0.74 (12.4)		-0.77 (- 1.0–0.58)
**Random–effect parameters**			
CL/*F*	0.25 (53.2)	2.9	0.25 (53.2)
Q/*F*	0.19 (45.7)	11.1	0.19 (45.7)
V_c_/*F*	0.23 (50.8)	8.1	0.22 (49.6)
V_p_ /*F*	0.17 (43)	13.9	0.18 (44.4)
Tlag	0.35 (64.7)	7.7	0.35 (64.7)
Tk_0_	0.11 (34)	23.7	0.1 (32.4)
Ka	0.55 (85.6)	19.6	0.51 (81.5)
**Residual variability**			
Additive residual error (ng/mL)	0.46 (8.9)		0.45 (0.37–0.64)
Proportional residual error	0.22 (5.4)		0.22 (0.19–0.24)

CL, clearance; Q, intercompartmental clearance; V_c_, central volume of distribution; V_p,_ peripheral volume of distribution; Tlag, absorption lag time; K_a_, absorption rate constant; TK_0_, zero–order absorption rate constant.

Coefficient of variation (CV%) for inter–individual variability was calculated using the following equation: $\sqrt{{e(\omega }^{2})-1}*100$

### Model validation

**Fig 5:**
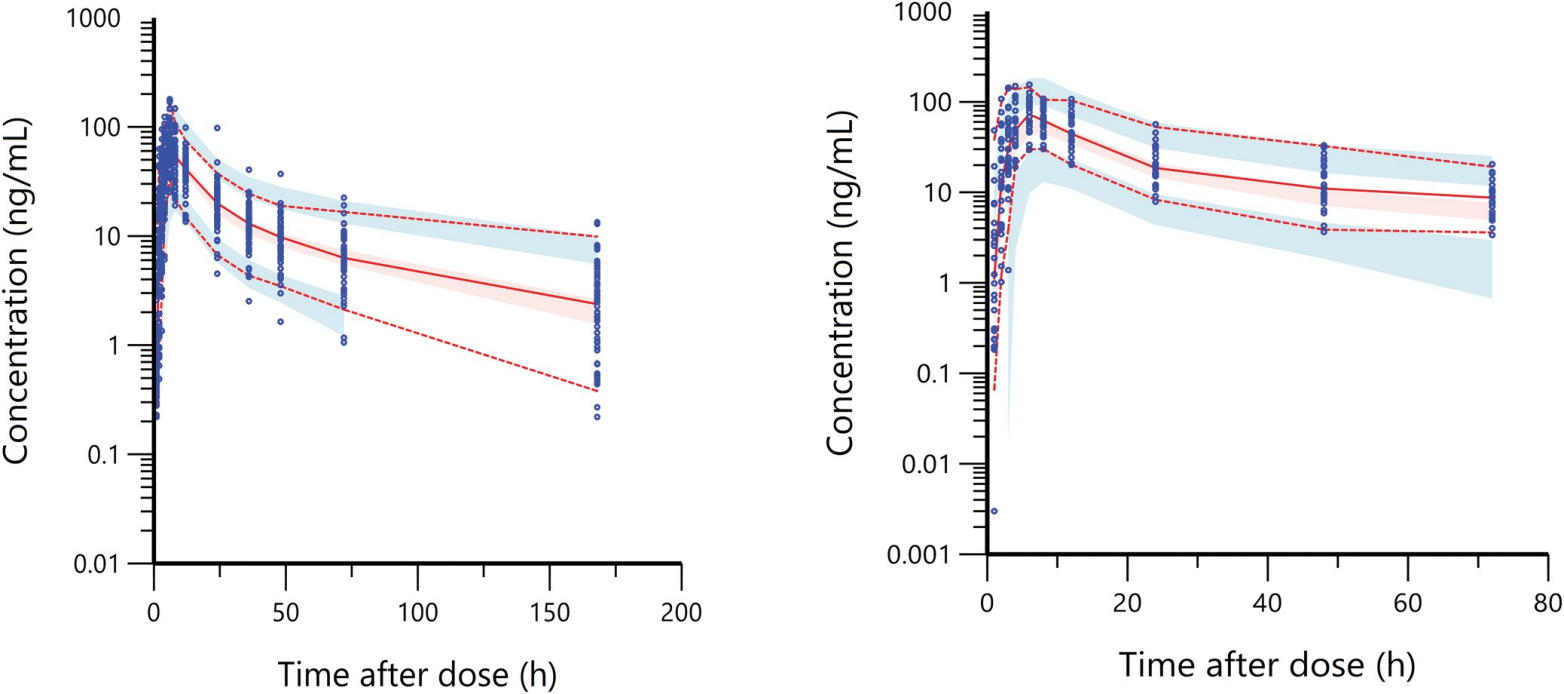
Visual predictive check (VPC) of the final model over the time from 0 and 168 hr (Left) and over the time from 0 and 72 hr for external validation (right), following IVM oral administration (n = 1000). A solid red line represents the 50^th^ percentile of observed data (blue dots). Dashed red lines represent the 5^th^ and 95^th^ percentiles of observed data. Shaded areas (blue and red) represent a 95% prediction interval of the 5^th^, 50^th^, and 95^th^ simulated data.

The median bootstrap values showed that the simulated data PK parameters were consistent with the estimated values predicted by the final model. PK parameters estimated by the final model were in the 2.5th and 97.5th percentiles of simulated data indicating the final model’s ability to estimate PK parameters with high precision **(Table 2)**. VPCs of the final model showed that the model predicted most observed concentrations within the 5th and 95th percentiles of simulated data indicating a good prediction performance of the final model **(Fig 5).** VPCs stratified by sex are shown in **S3 Fig.**

### External validation

The subject demographics of the external validation study are summarized in **S2 Table.** The VPCs of external validation showed that the final model predicted the observed concentrations from the external validation dataset with good prediction performance, similar to the validation results of original dataset **(Fig 5)**.

### Simulation

**Fig 6:**
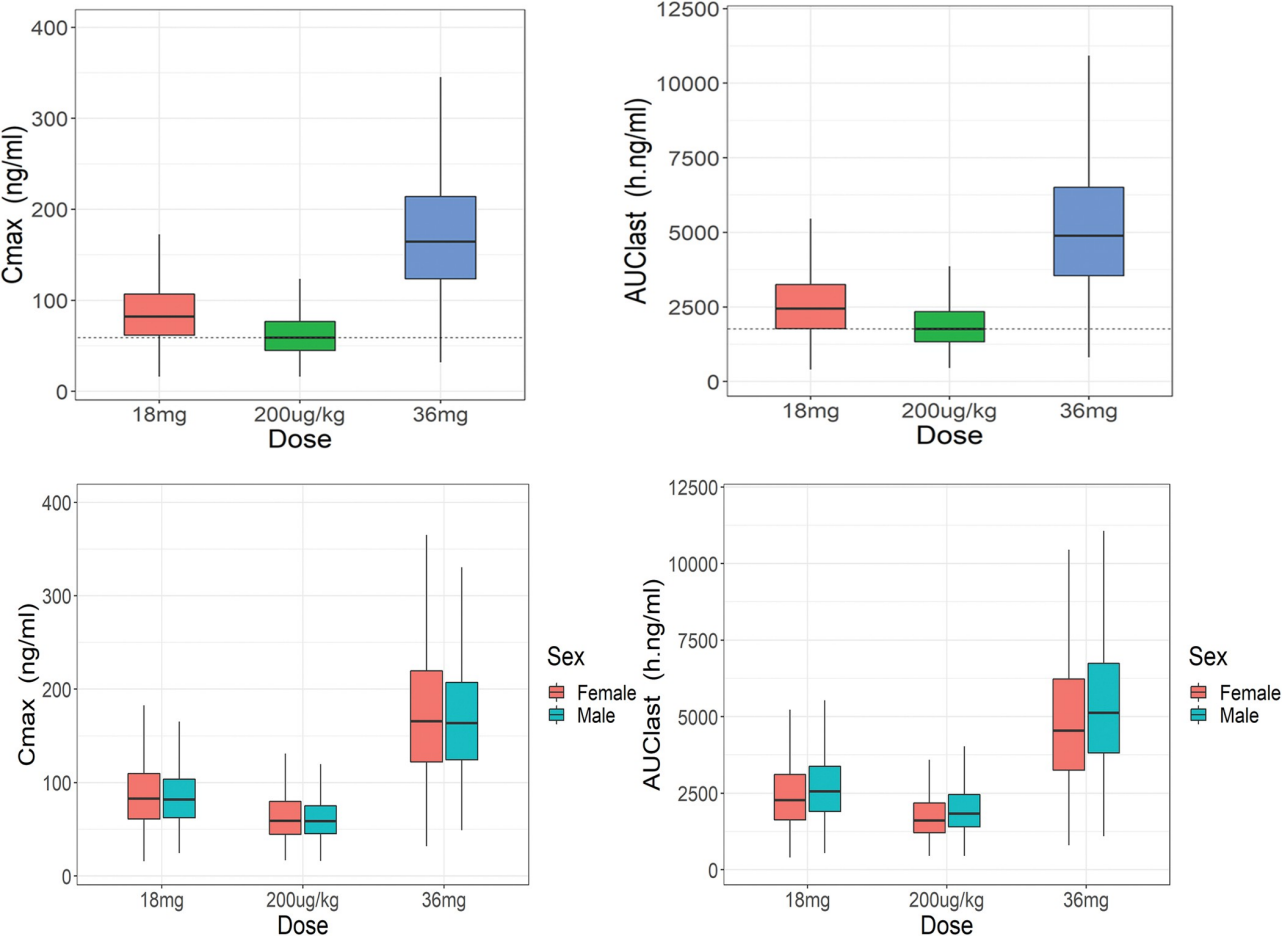
Comparison of model–based simulated IVM exposure (Cmax, AUClast) across simulated dosing groups (18 mg, 200 μg/kg, and 36 mg) (upper panels) and simulated exposure stratified by sex (lower panels). The box plot shows the median, 25th, and 75th quartiles.

The graphical comparison between predicted drug exposure associated with different dosing regimens is provided in **Fig 6**. The simulated population drug exposure followed three dosing groups showed a higher drug exposure with 36 mg dosing (median AUC_0-t_ and C_max_ of 4481 h*ng/ml, 164.47ng/ml, respectively) followed by 18 mg dosing (median AUC_0-t_ and C_max_ of 2240 h*ng/ml, 82.24 ng/ml, respectively) and a lower drug exposure with 200 μg/kg dosing (median AUC_0-t_ and C_max_ of 1759 h*ng/ml, 58.94 ng/ml respectively) **(S3 Table)**. As shown in **Fig 6**, the predicted overall drug exposure between males and females is not significantly different across all simulated dosing regimens. However, the median predicted AUC_0-t_ is slightly higher in males than females. The predicted AUC_0-t_ and C_max_ (median and range) of IVM for all three simulated dosing groups stratified by sex are shown in **S3 Table.**

## Discussion

Previous PopPK studies of IVM have used data from healthy volunteers to examine the effect of different covariates other than LF disease status on IVM drug exposure. This study’s population model was built that included patients with LF and healthy subjects. The final model indicates that the PK parameters of IVM are not affected by LF infection.

Final model estimates of CL/*F* and Vc/*F* were close to previous estimates (7.02 L/h and 138 L, respectively). The final model estimation of Vp/*F* was (424.33 L). Previously reported values for the PK of IVM in adults showed wide variability in reported Vp/*F* value with a range of (157–882 L) [12–14]. In these models, sex has not been shown to have a significant impact on Vp/*F*. In this model, Vp/*F* was lower by 53% in men than women. IVM is a lipophilic drug with a high volume of distribution that is widely distributed in the body after oral administration [22]. That females have a higher body fat percentage of body weight than males could explain why females have a higher peripheral volume of distribution of IVM than males [26]. In some previous population analysis studies of IVM, weight impacted the pharmacokinetic parameters of the drug. A population analysis of fixed-single dose (12 mg) of IVM in healthy volunteers by Duthaler, U., et al. shows that bodyweight influences the volume of the central compartment and clearance and was included in the model with allometric parameters scaling [12]. In population analysis by Kobylinski, K.C., et al, where IVM was dosed based on body weight as (200 μg/kg), the weight showed an insignificant impact on the model fitting when included with allometric scaling, but it was retained in the final model because of biological prior [14]. In this analysis, sex was the only significant covariate after applying the allometric scaling of body weight on all clearance and volume of distribution parameters. The study population shows a wide range of body weight and allometric scaling improved the model’s fit. Allometric scaling implemented in the final model can extend the model application to predict IVM exposure in pediatrics and teenage groups. However, IVM has not been approved yet to be used in MDA against LF in children weighing less than 15 kg [16]. In this analysis, the predicted drug exposure obtained from the model-based simulation showed a linear PK behavior across three simulated dosing groups, similar to previously reported results [14,17]. It was not significantly different based on sex. As shown in the simulation results, there was a higher drug exposure with the 18 mg fixed dose regimen than with the dose based on body weight at 200 μg/kg; however, the increase in drug exposure was relatively small. This finding may support using a fixed dose of 18 mg in MDA campaigns against LF in the adult population. As a matter of logistical concern in MDA, dosing IVM as a single fixed dose could be more practical than dosing based on body weight. The simulated drug exposure by the final model (AUC_0-t_) and (C_max_) was higher than the reported values in phase 1 clinical trial by Munoz, J., et al [18]. The possible explanation is the food effect on IVM exposure. In this model, the drug exposure was simulated based on taking IVM with food. The drug exposure of IVM is enhanced by food intake, especially by taking the drug with a fatty meal [27]. The final model can be used for dose informing studies, especially target attainment. However, future studies are needed to characterize the relationship between the plasma concentration of IVM and microfilaria (Mf) load to provide the required information for dose optimization to improve treatment effectiveness in LF. The lower limit of quantification value used in the analytical method allowed to include all available plasma concentrations in the model building process and improved the model’s ability to describe the elimination phase of the drug. In this analysis, external validation added an additional step to validate the prediction performance of the final model and confirm the result obtained by internal validation. Testing the prediction performance of models by external validation method using an external dataset provides a more rigorous way to test the prediction performance of the final model [28]. An extrapolation of the study findings should be limited to disease status and age groups evaluated (18–70 years). Future studies may be needed to characterize the effect of chronic infection of LF on IVM drug exposure. In chronic infection, the function of the lymphatic system can be severely compromised due to the effects of the disease on the function and architecture of lymphatic vessels [29]. High volume status resulting from filarial lymphedema, one of the complications seen in patients with advanced stage of the disease, could impact drug exposure. In this analysis, interindividual variability in absorption rate constant (Ka) is high (55%), and tested covariates have not explained it. There is high variability in IVM exposure reported in previous studies [6,18]. This variability could result from taking the drug with food. As a result, not standardizing food intake between subjects could be a reason for this high variability. We evaluated different absorption scenarios to improve the model’s fit since applying the lag time function alone could not provide a good fit to describe the early absorption phase. A zero-order dose input into the absorption compartment and the lag time function improved the data fitting. It may indicate the involvement of the lymphatic system in the gastrointestinal tract in IVM absorption after oral administration. In a modeling and simulation study to characterize lymphatic drug absorption, double peaks or shoulders appearing in the concentration-time profiles during the absorption phase were associated with adding a lag time function to the lymphatic-based absorption model [30]. The presented analysis experienced a similar pattern, where some individual plasma concentration-time curves showed shoulders appearing before C_max_ during the absorption phase. The lymphatic system can play a role in the PK of the drugs, especially the drug absorption after oral administration. Drug absorption through the intestinal lymphatic system has been identified as a possible pathway for drug absorption into systemic circulation as another route to portal absorption [30]. Physicochemical characteristics, such as having high lipid solubility and a log P value more than five, can determine the ability of the drug to be absorbed more likely by the lymphatic system in the intestines than by portal circulation[15]. The physicochemical properties of IVM make it a possible candidate for lymphatic absorption as it is a lipophilic drug with a high molecular weight and a log P value > 5. Future studies in animal models are needed to identify the extent of IVM’s intestinal lymphatic absorption, which could increase our understanding of IVM PK and explain the high variability in systemic drug exposure observed following the oral administration of IVM.

There are several limitations to this study. First, a relatively small sample size was used, which may have impacted the ability to capture the possible effects of other factors on IVM PK. The demographic data collected did not include height, limiting our ability to further characterize the influence of body size and composition on IVM disposition. Height data would have allowed for including additional weight-related measurements, such as body mass index (BMI), lean body weight (LBW), and fat-free mass (FFM). Those measurements could have served as additional covariates to explain drug exposure variability by implementing different allometric scaling on FFM and LBW. Further studies evaluating the influence of body size and composition on IVM PK are warranted. Another limitation of this analysis is that we did not investigate the possibility that genetic polymorphisms might influence the variability in IVM drug exposure. IVM is a substrate for Cytochrome P450 (CYP) enzymes and multidrug resistance (MDR1) genes, and its pharmacokinetics could be impacted by polymorphisms associated with those genes [22,31,32]. Future PopPk studies, including genetic polymorphisms as a covariate, are needed to investigate this impact on IVM drug exposure.

**In conclusion**, We have developed a PopPK model for IVM using data obtained from LF patients and healthy volunteers. This was the first PopPK study of IVM to include patients infected with *Wuchereria bancrofti*. The model validation findings indicate the robustness and excellent prediction performance. It can be used for future dose simulation studies of IVM in treating LF.

## Supporting information

S1 Table Tested Models comparison. (DOCX)

S2 Table Subjects demographics of the validation dataset. (DOCX)

S3 Table Simulated median values for (AUC 0-last, Cmax) across simulated dosing groups. (DOCX)

S1 Fig The goodness of fit plots of Base Model. Observed plasma concentrations of IVM versus individual predicted concentration (A) and population predicted concentration (B), conditional weighted residuals (CWRES) versus population predicted concentration (C), versus (Time after dose) (D). Black lines in (A) and (B) are the line of identity. The blue line in (C) and (D) represent the locally weighted scatterplot smoothing line (LOWESS); dashed black lines are margins (y = ±2) of outliers.
(TIF)

S2 Fig ETA Box Plots. Box plots show the impact of sex (0 = Female, 1 = Male) and LF infection status (0 = Uninfected, 1 = infected) on Eta values of IVE Pk parameters. Box plots and whiskers represent the distribution of the value of IVE Pk parameters between covariates. CL (Central clearance), Q (peripheral clearance), V (volume of distribution of central compartment), V_2_ (volume of distribution of peripheral compartment)
(TIF)

S3 Fig Visual predictive check (VPC) stratified by sex (left = Female, right = Male) of the final model over the time from 0 and 168 hr. following IVE oral administration (n = 1000). Solid red lines represent the 50^th^ percentile of observed data (blue dots). Dashed red lines represent the 5^th^ and 95^th^ percentiles of observed data. Shaded areas (blue and red) represent a 95% prediction interval of the 5^th^, 50^th^, and 95^th^ simulated data.
(TIF)

S1 Text Phoenix NLME (PML) of the final model. (DOCX)
