# Supplementary material for: Population pharmacokinetic model of ivermectin in mass drug administration against lymphatic filariasis
Source: PLoS Negl Trop Dis. 2023 Jun 1;17(6):e0011319. doi: 10.1371/journal.pntd.0011319 (PMC10234547; doi:10.1371/journal.pntd.0011319)
Supplement: S1 Table — (DOCX) [file pntd.0011319.s001.docx]

| **Model** | **Model Description** | **OFV** | **ΔOFV** |
| --- | --- | --- | --- |
| **Base Model** | **One compartments (First order absorption)**  **- (Additive error)**  **- (Proportional error)**  **- (Additive + Proportional)**  **Two compartments (First order absorption)**  **- (Additive error)**  **- (Proportional error)**  **- (Additive + Proportional)**  **Two compartments (First order absorption) + Time lag function on absorption (Tlag)**  **- (Additive+ Proportional)**  **Two compartments (simultaneous zero- and first-order absorption)**  **- (Additive+ Proportional)**  **Two compartments (sequential zero- and first-order absorption) +Tlag**  **- (Additive+ Proportional)**  **Two compartments (Transit absorption)**  **- (Additive+ Proportional)**  **Two Compartments (first order absorption with zero order input) +Tlag (including allometric scaling)**  **- (Additive+ Proportional)** | **5803**  **5407**  **5314**  **5794**  **5064**  **5063**  **4689**  **4439**  **4325**  **4280**  **4269 (Selected Base Model)** |  |
| **Final Model** | **Final Model with sex on Vp**  **- (Diagonal covariance)**  **- (Non-diagonal covariance) (Omega block)** | **4241**  **4125 (Final Model)** | **28**  **144** |
| **Tlag, Time lag; V_p,_ peripheral volume of distribution**  **OFV, objective function value**  **ΔOFV, reduction in Objective Function Value from the selected base model** | | | |

**S1 Table. Tested Models comparison**
