# Supplementary material for: Population pharmacokinetic model of ivermectin in mass drug administration against lymphatic filariasis
Source: PLoS Negl Trop Dis. 2023 Jun 1;17(6):e0011319. doi: 10.1371/journal.pntd.0011319 (PMC10234547; doi:10.1371/journal.pntd.0011319)
Supplement: S2 Table — (DOCX) [file pntd.0011319.s002.docx]

**S2 Table. Subjects demographics of the validation dataset**

| **Demographics Variables** | **Values** |
| --- | --- |
| **Sex**  No. of males (%)  No. of females (%)  **Age (Years)**  Median(range)  **Bodyweight (Kg)**  Median(range)  **No. of plasma samples** | **12 (48%)**  **13 (50%)**  **27.5 (18-59)**  **62 (46-93)**  **286** |
