# Supplementary material for: Population pharmacokinetic model of ivermectin in mass drug administration against lymphatic filariasis
Source: PLoS Negl Trop Dis. 2023 Jun 1;17(6):e0011319. doi: 10.1371/journal.pntd.0011319 (PMC10234547; doi:10.1371/journal.pntd.0011319)
Supplement: S3 Table — (DOCX) [file pntd.0011319.s003.docx]

**S3 Table**. Simulated median values for (AUC 0-last, Cmax) across simulated dosing groups.

| **Simulated**  **Dosing regimen** | **Median (Range)** | | |
| --- | --- | --- | --- |
|  | **All subjects** | **Male** | **Female** |
| **200ug/kg**  **AUC0-last** (h*ng/ml)  **Cmax** (ng/ml) | 1759 (455-8368)  58.94 (16.34-261.3) | 1834 (455-8368)  58.73 (16.34-234) | 1612 (457-5712)  59.23(16.85-261.3) |
| **18 mg**  **AUC0-last** (h*ng/ml)  **Cmax** (ng/ml) | 2240 (402-12552)  82.24 (16.02-391.95) | 2563 (546-12552)  81.86 (24.52-351.04) | 2272 (402-8568)  82.88 (16.02-391.95) |
| **36 mg**  **AUC0-last** (h*ng/ml)  **Cmax** (ng/ml) | 4881 (805-25105)  164.47 (32.03-783.89) | 5126 (1092 -25105)  163.72 (49.03-702.8) | 4545 (805-17136)  165.76 (32.03-783.89) |
