# Supplementary figures and images for: Population pharmacokinetic model of ivermectin in mass drug administration against lymphatic filariasis
Source: PLoS Negl Trop Dis. 2023 Jun 1;17(6):e0011319. doi: 10.1371/journal.pntd.0011319 (PMC10234547; doi:10.1371/journal.pntd.0011319)

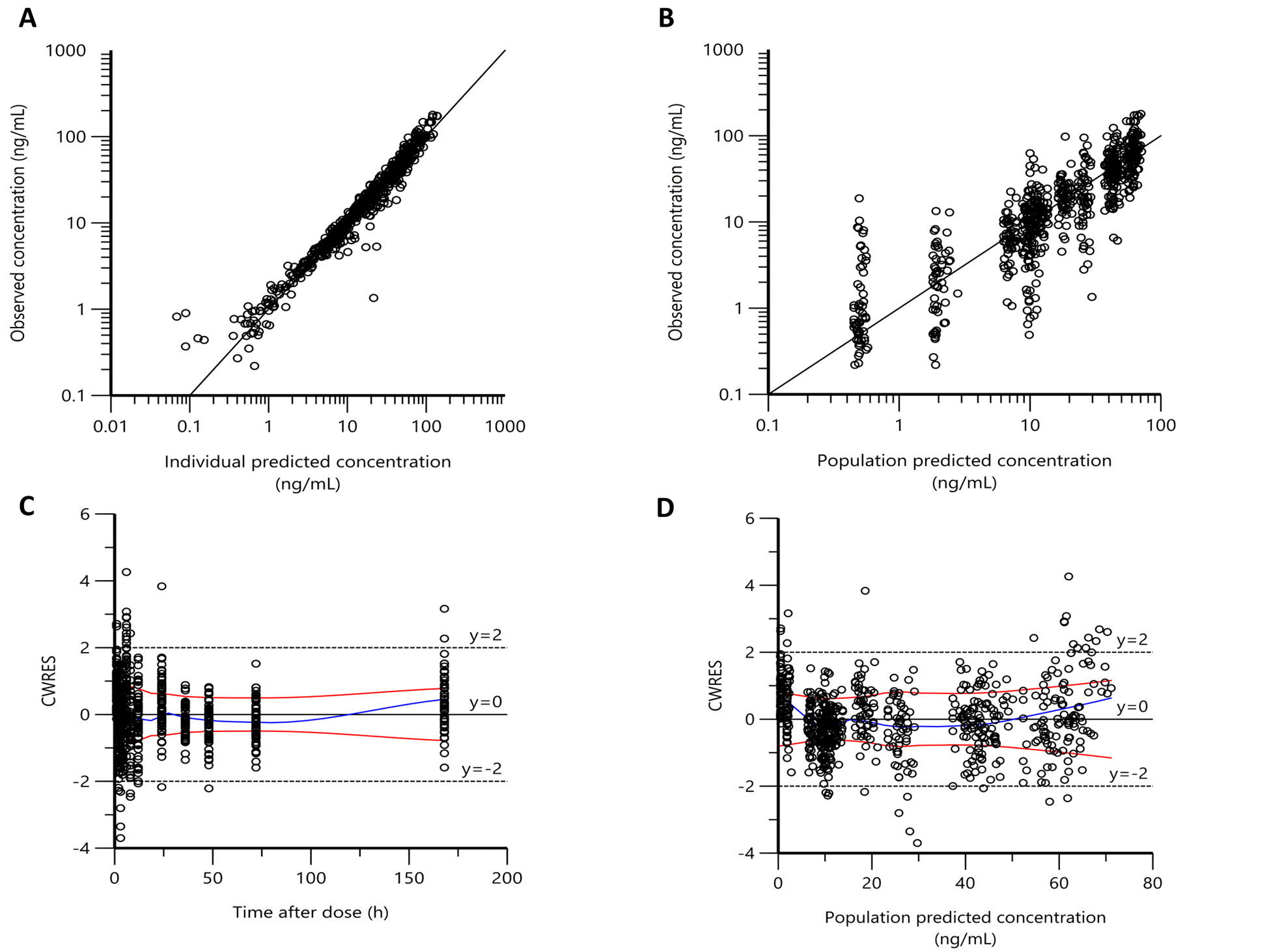

Supplement: S1 Fig — Observed plasma concentrations of IVM versus individual predicted concentration (A) and population predicted concentration (B), conditional weighted residuals (CWRES) versus population predicted concentration (C), versus (Time after dose) (D). Black lines in (A) and (B) are the line of identity. The blue line in (C) and (D) represent the locally weighted scatterplot smoothing line (LOWESS); dashed black lines are margins (y = ±2) of outliers. (TIF) [file pntd.0011319.s004.tif]

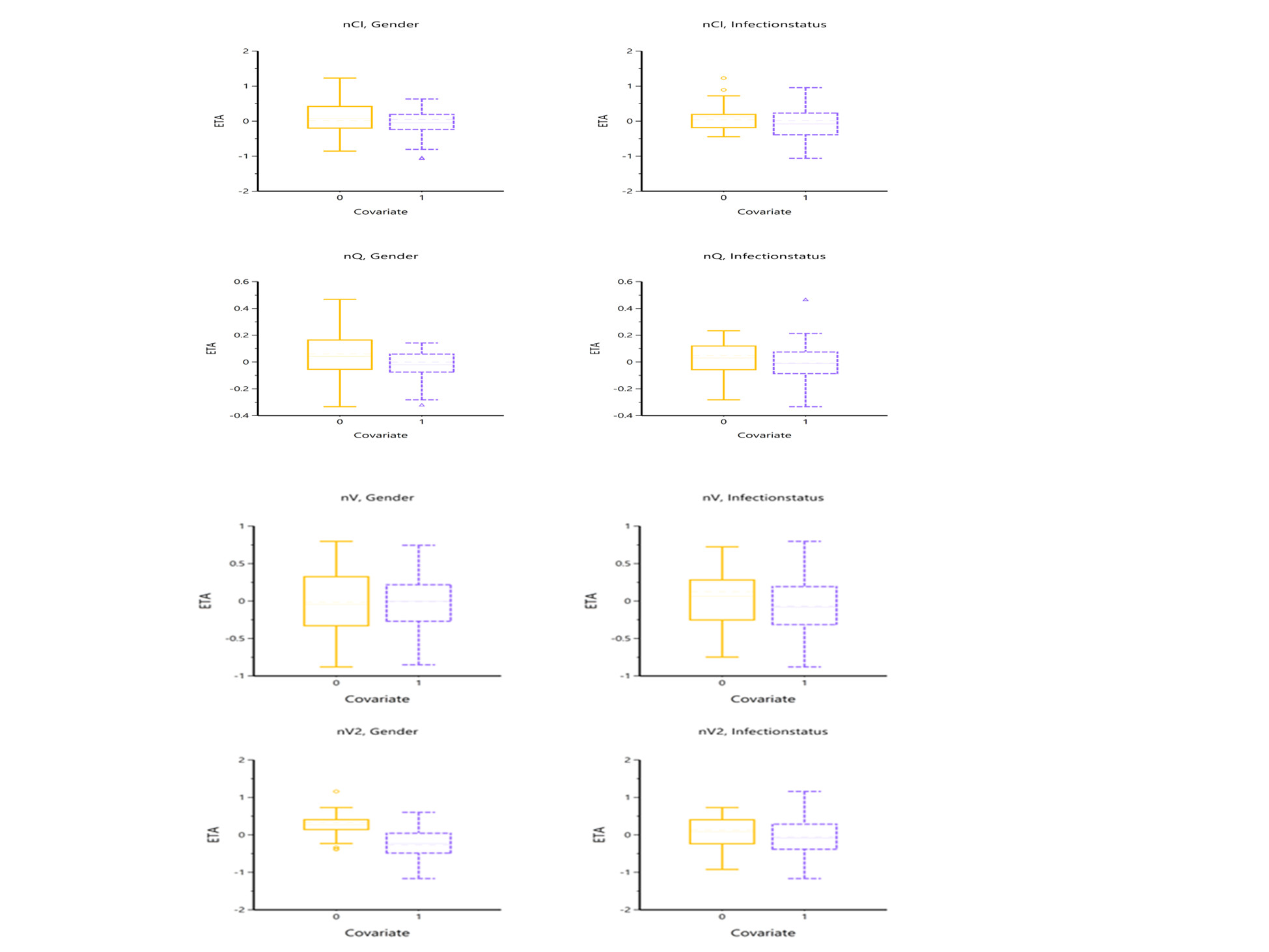

Supplement: S2 Fig — Box plots show the impact of sex (0 = Female, 1 = Male) and LF infection status (0 = Uninfected, 1 = infected) on Eta values of IVE Pk parameters. Box plots and whiskers represent the distribution of the value of IVE Pk parameters between covariates. CL (Central clearance), Q (peripheral clearance), V (volume of distribution of central compartment), V2 (volume of distribution of peripheral compartment) (TIF) [file pntd.0011319.s005.tif]

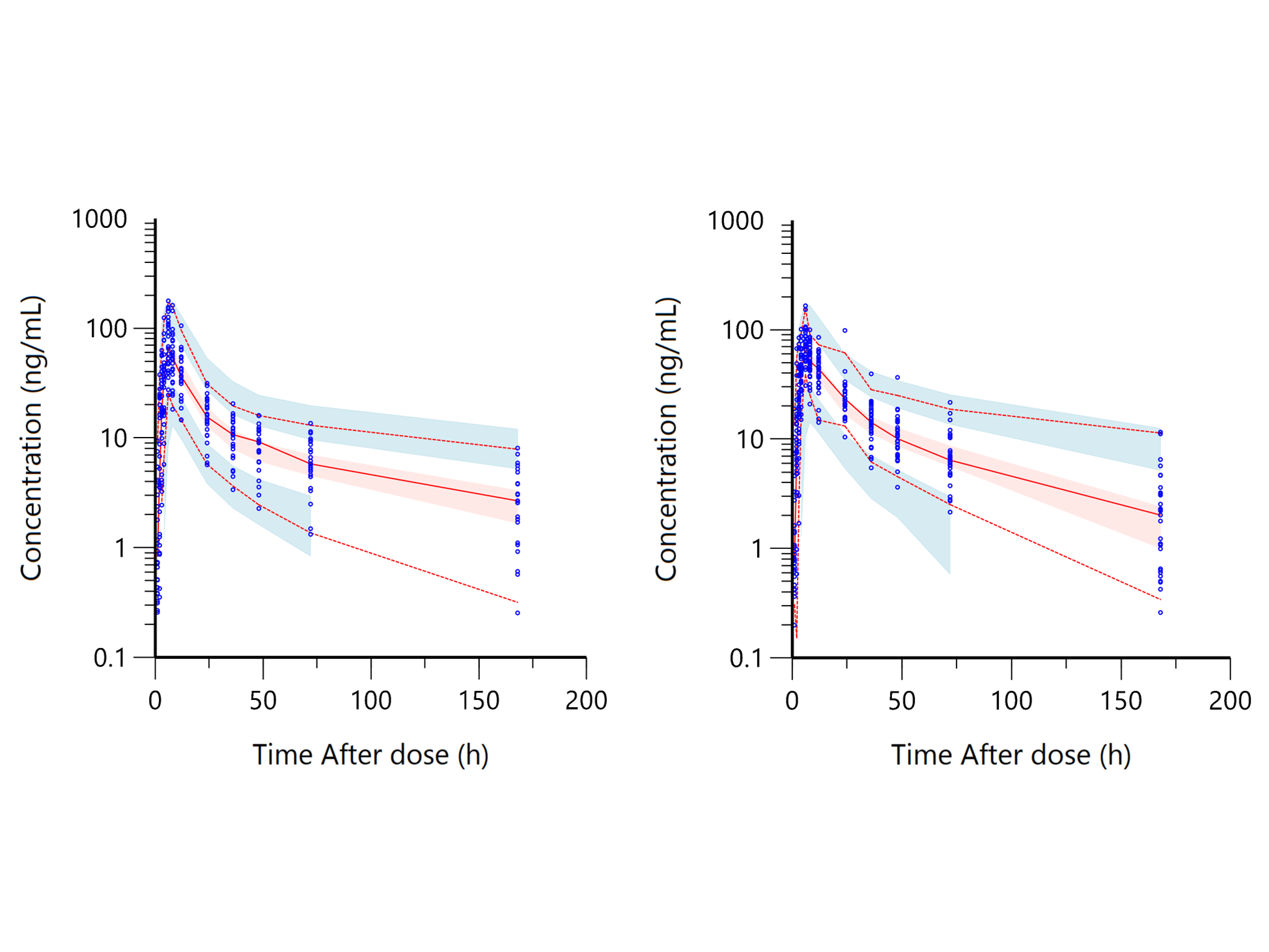

Supplement: S3 Fig — Solid red lines represent the 50th percentile of observed data (blue dots). Dashed red lines represent the 5th and 95th percentiles of observed data. Shaded areas (blue and red) represent a 95% prediction interval of the 5th, 50th, and 95th simulated data. (TIF) [file pntd.0011319.s006.tif]
