# Supplementary material for: Population pharmacokinetic model of ivermectin in mass drug administration against lymphatic filariasis
Source: PLoS Negl Trop Dis. 2023 Jun 1;17(6):e0011319. doi: 10.1371/journal.pntd.0011319 (PMC10234547; doi:10.1371/journal.pntd.0011319)
Supplement: S1 Text — (DOCX) [file pntd.0011319.s007.docx]

**S1 Text. Phoenix NLME (PML) of the final model**

test(){

cfMicro(A1, Cl / V, Cl2 / V, Q / V2, first = (Aa = Ka))

dosepoint(Aa, tlag = Tlag,duration = TK0 )

C = A1 / V

error(CEps = 0.46110892757607)

observe(CObs = C + CEps * sqrt(1 + C^2 * (CMultStdev/sigma())^2))

stparm(Ka = tvKa * exp(nKa))

stparm(V = tvV * (Weight/mean(Weight))^dVdWeight * exp(nV))

stparm(V2 = tvV2 * (Weight/mean(Weight))^dV2dWeight * exp(dV2dSex1*(Sex==1)) * exp(nV2))

stparm(Cl = tvCl * (Weight/mean(Weight))^dCldWeight * exp(nCl))

stparm(Q = tvQ * (Weight/mean(Weight))^dQdWeight * exp(nQ))

stparm(Tlag = tvTlag * exp(nTlag))

stparm(TK0 = tvTK0* exp(nTK0))

stparm(CMultStdev = tvCMultStdev)

fcovariate(Sex())

fcovariate(Weight)

fixef(tvKa = c(, 0.717722387163157, ))

fixef(tvV = c(, 138071.74209207, ))

fixef(tvV2 = c(, 424334.354384267, ))

fixef(tvCl = c(, 7026.63375480997, ))

fixef(tvQ = c(, 9113.17023736889, ))

fixef(tvTlag = c(, 0.756753148154855, ))

fixef(tvTK0 = c(, 3.73958407653678, ))

fixef(tvCMultStdev = c(, 0.228212446759966, ))

fixef(dVdWeight = c(, 1, ))

fixef(dV2dSex1(enable=c(17)) = c(, -0.74325100598213, ))

fixef(dV2dWeight = c(, 1, ))

fixef(dCldWeight = c(, 0.75, ))

fixef(dQdWeight= c(, 0.75, ))

ranef(block(nV, nCl, nKa, nV2, nQ, nTlag, nTK0) = c(0.23, 0.25, 0.55, 0.17, 0.19, 0.35, 0.11))

}
